# A systematic review and meta-analysis of surgical morbidity of primary versus patch repaired congenital diaphragmatic hernia patients

**DOI:** 10.1038/s41598-021-91908-7

**Published:** 2021-06-16

**Authors:** Kim Heiwegen, Ivo de Blaauw, Sanne M. B. I. Botden

**Affiliations:** Division of Pediatric Surgery, Department of Surgery, Radboudumc-Amalia Children’s Hospital, route 618, PO Box 9101, 6500 HB Nijmegen, The Netherlands

**Keywords:** Neonatology, Paediatric research

## Abstract

Large studies comparing the surgical outcome of primary versus patch repair in congenital diaphragmatic hernia (CDH) patients are rare. This study aims to evaluate the incidence of surgical complications in both types of CDH repair. PubMed, EMBASE, Cochrane and Web of Science were searched for peer-reviewed articles. Studies on CDH between 1991 and August 2020 were systematically screened and meta-analyses were performed. Primary outcomes of this review were: haemorrhage, chylothorax, recurrences and small bowel obstruction (SBO). A total of 6436 abstracts were screened, after which 25 publications were included (2910 patients). Patch repaired patients have a 2.8 times higher risk on developing a recurrence (20 studies) and a 2.5 times higher risk on developing a chylothorax (five studies). Moreover, they have a two times higher risk on developing a SBO. No studies could be included that evaluated the incidence of surgical haemorrhage between these patients. Although the quality of the studies was relatively low, patch repaired patients have a higher risk on developing a recurrence, chylothorax and small bowel obstruction. Large prospective studies are required to adjust for severity of disease, to reveal the true causative factors in order to minimize the risk on these surgical complications in both types of patients.

## Introduction

Treatment of congenital diaphragmatic hernia (CDH) patients remains challenging. Surgical repair of CDH is required in order to relocate the herniated organs from the thoracic cavity to the abdomen and close the diaphragmatic defect. This repair is mostly performed by an open procedure, while there is ongoing discussion concerning the advantages and disadvantages of minimally invasive surgery^1^. Minimally invasive surgery has been associated with lower use of patch and higher incidence of recurrences^2–4^. The majority of the defects are repaired primarily (60–70%), whereas the more severe cases (and largest defects) are repaired with the use of a patch^5^. Different types of patch materials have been used for closure of diaphragmatic defects. While some studies state that non-absorbable prosthetic patches, mainly PTFE (polytetrafluoroethylene), are preferred^4,6^, others prefer absorbable patches, such as collagen, or muscle-flap repair^7^ and others do not show a preference for type of material^8^. Although not many comparative studies evaluate primary versus patch repair, some complications have been highly associated with patch repair, such as chylothorax, which could lead to respiratory failure if not treated promptly^9–11^. Moreover, previous cohort studies have reported that patch repair is associated with higher rates of recurrences^9,12^. Because there is a lack of prospective and large studies on type of repair, this article provides a systematic review and meta-analysis to evaluate current evidence on the challenging topic of type of repair in CDH and the concurrent risk on surgical complications.

## Methods

### Search

A systematic literature search was performed in the databases PubMed, Web of Science, Cochrane and Embase for studies published between 1991 and August 2020 by the first author and a librarian. The studies were screened according to the Preferred Reporting Items for Systematic Reviews and Meta-Analysis (PRISMA) flow chart^13^. The search terms used were based on the subject (‘’congenital diaphragmatic hernia’’) and procedure (‘’patch repair’’ and ‘’primary repair’’). Search terms were restricted to title, abstract and keywords. When series from the same institution were found, the most recent study was included. Two authors (Sanne Botden, SB and Kim Heiwegen, KH) screened titles, abstracts and eventually full-text articles on inclusion and exclusion criteria independently. Final decision was based on discussion and consensus between these two authors. This review was registered on the international prospective register of systematic reviews (PROSPERO: CRD42019123189)^14^.

### Selection criteria for included studies

Studies comparing primary repair versus patch repair in neonates with CDH, reporting on minimally one of the outcomes were included. Exclusion criteria were studies focusing on Extracorporeal Membranous Oxygenation (ECMO) particularly, review articles, case reports, opinion papers, case series with ≤ 5 patients in both compared groups and animal studies.

### Data abstraction from included studies

The following data was extracted from each study separately; study characteristics (author names, year of publication, study design, study period, sample size and investigated type of repair), patient characteristics (age, gender, APGAR-score, side of defect, size of defect), method of reassigning patients to type of repair, peri-operative characteristics (surgical approaches in both groups, type of repair) and surgical morbidity. Surgical morbidity was stated as outcomes needing possible surgical intervention, which included the postoperative complications haemorrhage and recurrence, but also chylothorax and small bowel obstruction (SBO). The data was double checked by two authors independently (SB and KH).

### Methodological quality

Two authors (SB and KH) independently assessed all articles on methodological quality according to the Grading of Recommendations Assessment, Development, and Evaluation (GRADE) system^15^. The quality of evidence could be rated in four scores; high, moderate, low or very low. Downward rating was based on risk of bias, inconsistency, indirectness, imprecision (using optimal information size: OIS) and publication bias. The Risk of Bias in Non-Randomized Studies- of Interventions (ROBINS-I) tool was used to score studies on risk of bias, because no randomized studies were expected on this subject (Supplementary Information)^16^.

### Statistical analyses

The statistical analyses were performed using Review Manager (RevMan) 5.3 Software, provided by the Cochrane Collaboration (Oxford, England). Categorical data were analyzed using the Mantel–Haenszel method*.* Heterogeneity between studies was assessed using I^2^ (percentage of the variability in effect estimates due to heterogeneity rather than sampling error), with percentages of 30% considered low, 30–50% as moderate and ≥ 50% as considerable heterogeneity^17,18^. The random-effect model was used to assess the combined effect. Forests plots were used to graphically display risk ratios for categorical variables, including their 95% confidence interval. Publication bias was assessed for outcomes including more than ten studies by visual interpretation of a funnel plot (developed with RevMan). Sensitivity analyses were planned with exclusion of serious and or critical risk of bias according to ROBINS-I tool. Subgroup analyses, were performed on approach of repair (open versus minimally invasive (MIS) and on exclusion of patients treated before 2000 (because treatment strategies might have been different).

## Results

### Study selection

**Figure 1:**
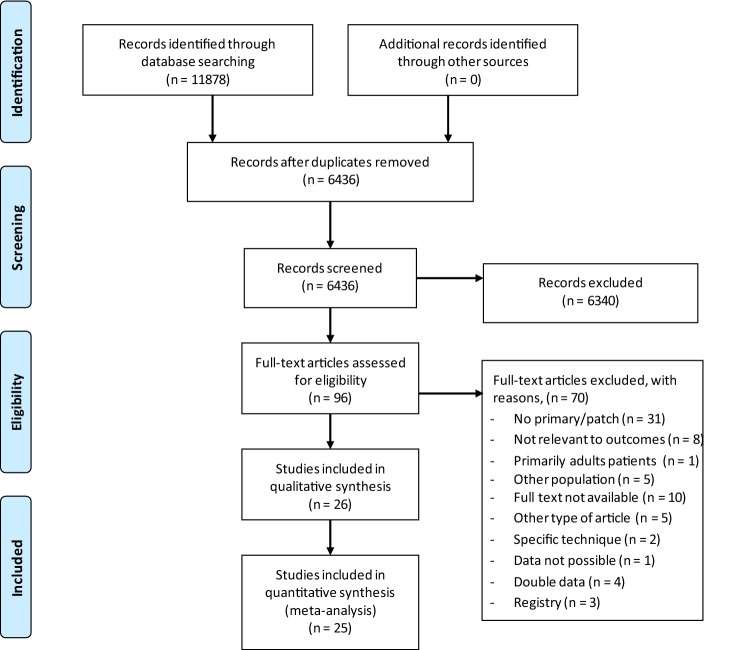
PRISMA flow diagram^13^.

In total, 11,878 studies were identified by database searching, which is presented in the PRISMA Flow Diagram (Fig. 1). Manual screening did not lead to any new articles. Title and abstract screening were performed in 6436 studies, of which 96 remained eligible for full-text assessment. Another 71 were excluded due to several reasons (see Fig. 1), leading to inclusion of 25 studies. Studies which included registry data were excluded, because this could lead to double inclusion of patients^19–21^. For hernia recurrences 20 cohort studies were included, ten for SBO and five for chylothorax^2,4,10,12,22–42^. No studies were found reporting on rate of haemorrhage. By attempts to contact authors, additional data could not be obtained, leading to exclusion of one study^43^.

### Characteristics of study

Study characteristics of the included quantitative studies are shown in Table 1. All included studies were cohort studies on CDH patients specifically (n = 2910). The mean rate of primary versus patch repaired patients was 57% (n = 1704) versus 43% (n = 1254). Different types of patch were used, although most studies (18/25) included used non absorbable patches such as PTFE (n = 16) or Polyester (n = 6). Collagen patches were used in five patients. The division of open versus MIS approach in the thirteen studies, which presented data on surgical approach, was 72.8% versus 27.2%, while the remaining studies did not specify approach of repair specifically. There was a wide range of duration of follow-up, which ranged from four months till eleven years and was not specified in some of the studies.

**Table 1:** Study characteristics.

Reference (first author)	Period	No. of patients	Type of repair		Type of patch	Open/MIS**	Complications, n (%)			Follow-up
			Primary	Patch*			Recurrence	SBO	Chylothorax	
Al-Iede	2000–2013	85	65	20	PTFE/Dacron	71/14	11 (12.9)			2y
Aydin	2005–2016	119	28	91	PTFE	119/12(excl.)	6 (5.0)			5ya
Costerus	2008–2012	109	42	67	PTFE	34/75	16 (14.8)			1y
Fisher	1990–2006	238	128	110	PTFE	231/7	24 (10.1)			
Gander	2006–2010	45	17	28	PTFE	19/26	6 (17.1)			14m^a^
Gonzalez	1997–2008	152	96	56	Not specified	137/15			10 (6.6)	
Guner	2004–2008	15	10	5	Dacron	0/15	3 (20)			4–14 m
Hanekamp	1990–2000	89	48	41	Not specified	Not specified			9 (10)	
Jancelewicz (To)	2000–2011	157	115	42	PTFE/SIS	129/28	23 (15)	12 (8)		
Jancelewicz (SF)	2000–2008	99	42	57	PTFE/SIS/flap	94/5	30 (30.3)	13 (13.1)		4.7y^a^
Janssen	2000–2014	112	77	35	PTFE	Not specified	8 (7.1)	22 (19.6)		7.3y^b^
Jawaid	1990–2010	118	81	37	PTFE/SIS	118/0	2 (1.8)	2 (1.8)		8.6y^a^
Kamata	1986–2000	33	18	15	Not specified	Not specified	4 (12)			11.4^b^
Kamiyama	1981–2008	198	109	89	PTFE	Not specified			11 (5.6)	
Keijzer	2006–2008	46	18	28	PTFE	29/17	4 (15)			1y^c^
Laituri	1994–2009	155	101	54	PTFE/Dacron/SIS/Alloderm	Not specified	23 (14.8)	20 (13)		
Lund	Not specified	33	23	10	PTFE	Not specified	2 (6.1)			32m^b^
Mills	2006–2010	230	159	71	Not specified	Not specified			11 (4.5)	
Nagata	2006–2010	180	112	68	Not specified	180/0	21 (11.6)			3y^c^
Riehle	1993–1994	125	79	46	PTFE	Not specified	2 (1.6)	6 (4.8)		27m^a^
St Peter	1994–2004	81	57	24	NA mesh, SIS	Not specified	10 (12.3)	8 (9.9)		7.95y^a^
Suply	2000–2016	203	96	107	PTFE, fibrine	55/148	14 (6.9)	8 (3.9)		
Tsai	1999–2010	149	75	74	PTFE	136/12	7 (8.6)	5 (3.4)		2y^a^
Yokota	1995–2013	74	49	25	‘Artificial patch’	74/0	8 (10.8)	13 (17.6)		50m^a^
Zavala	2003–2009	65	29	36	Not specified	Not specified			7 (10.8)	

*Patch including flap.

**Open: abdominal or thoracotomy, MIS: Thoracoscopic or laparoscopic (Gonzalez, Jancelewicz ((To), 1)).

^a^Median, ^b^Mean, ^c^Minimum, PTFE = polytetrafluorethylene, m = month(s), NA = non-absorbable, No. = total number, SIS = biological mesh (f.e. Surgisis®) y = year(s).

### Methodological quality

The ROBINS-I classification of the included studies are separately presented in the supplementary material. Three multicenter studies were found, of which two were national registries (Canada and Japan)^23,36,37^. Ten studies were classified as serious or critical risk of bias, mostly due to unequal follow-up and selection bias. Moreover, confounding for disease severity factors, such as size of the defect or Apgar scores, was not possible or not present in most of the studies.

### Haemorrhage

None of the included studies reported on surgical haemorrhage in primary versus patch repaired patients. One study by Brant et al.^44^ comparing primary versus patch and abdominal muscle flaps in CDH patients reported on haemorrhages, but was excluded from this review due to lack of required data. They reported that two patients in the prosthetic group had haemorrhages; one patient after day two, while the other required two reoperations for abdominal clot evacuation. None of the patients with a muscle flap had reported bleeding, though significance was not calculated.

### Chylothorax

**Figure 2:**
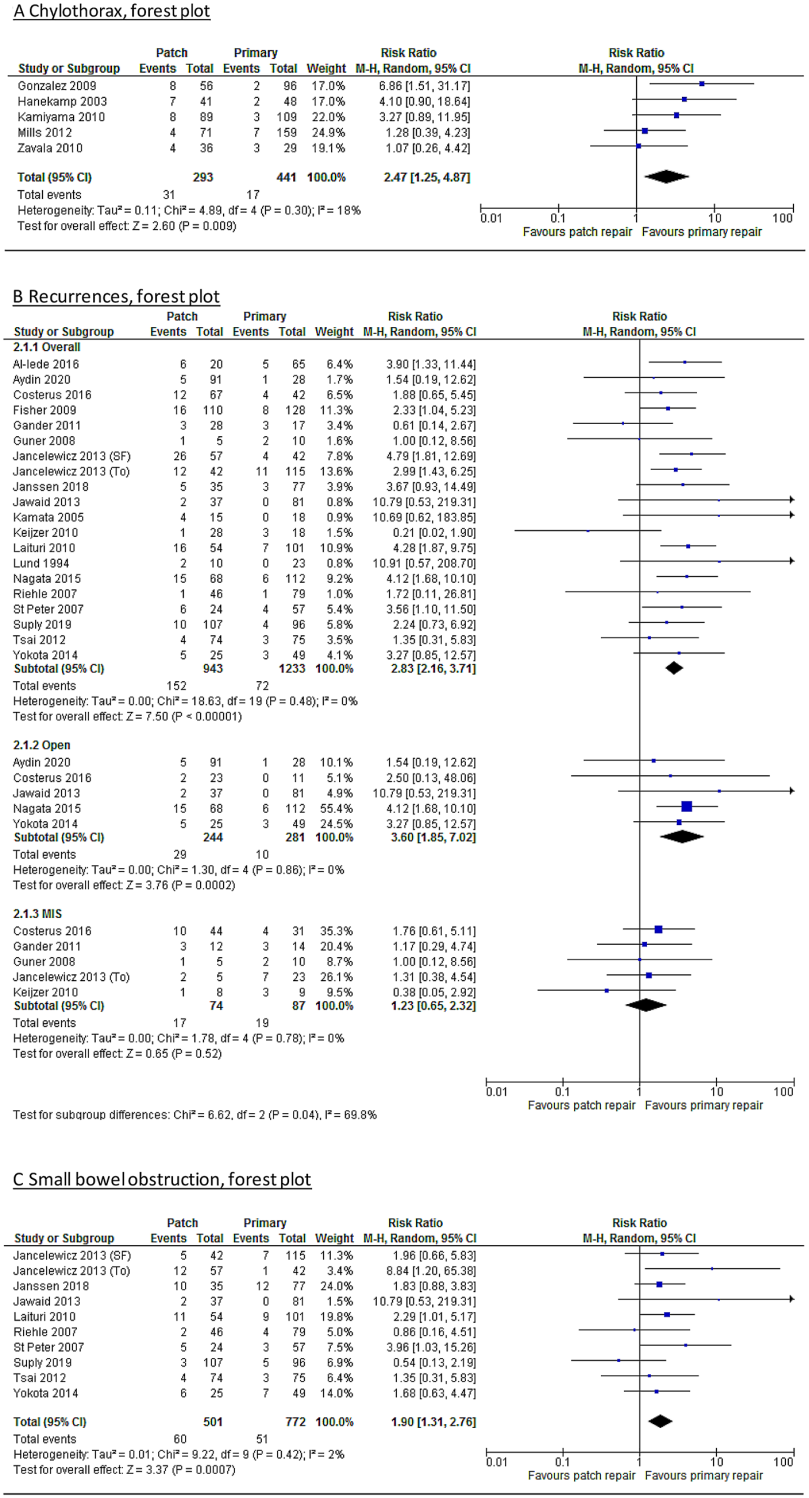
(**A**) Forest plot – chylothorax. (**B**) Forest plot—recurrence. (**C**) Forest plot—small bowel obstruction.

A meta-analysis on the post-operative complication chylothorax is shown in Fig. 2A. Five studies were included, which showed that the patch repaired patients have an increased risk (risk ratio = 2.47 [1.25–4.87]) on developing a chylothorax compared to primary repaired patients. Hanekamp et al.^27^ included solely ECMO patients. Therefore, a subanalysis was performed without this study in which the difference remained statistically significant (risk ratio = 2.24 [1.00–5.04]). A sub analysis excluding patients treated before 2000 was not possible, as only two studies included solely patients after 2000, of which one was scored as critical bias.

### Recurrence

In total, 20 studies reported on recurrence rate for primary and patch repaired patients separately. The overall recurrence rate was 16.2% versus 5.8%. Patients requiring patch repair have a 2.83 [2.16–3.71] greater risk on developing a recurrence (Fig. 2B). After exclusion studies with patients treated before 2000 (n = 9), the difference remained significant 2.47 [1.62, 3.77]. Sub-analyses were performed, if possible, on thoracoscopic versus open repaired patients. This showed that the difference for recurrence did not remain significant in minimally invasive repaired patients (RR 1.23 [0.65–2.32]). For the open repaired patients this was even higher than for both approaches combined (RR 3.60 [1.85–7.02]) (Fig. 2B).

### Small bowel obstruction

In Fig. 2C the meta-analysis of the ten studies reporting on SBO is presented. The mean rate of SBO was 6.6% for primary repaired patient versus 12% of the patch repaired patients. Meta-analysis also shows that patch repaired patients are at greater risk of developing a SBO (risk ratio = 1.90 [1.31–2.76]). This remained significant in the subanalysis of the patients treated after 2000 (n = 5), risk ratio 2.03 [1.01–4.07].

### Publication bias

**Figure 3:**
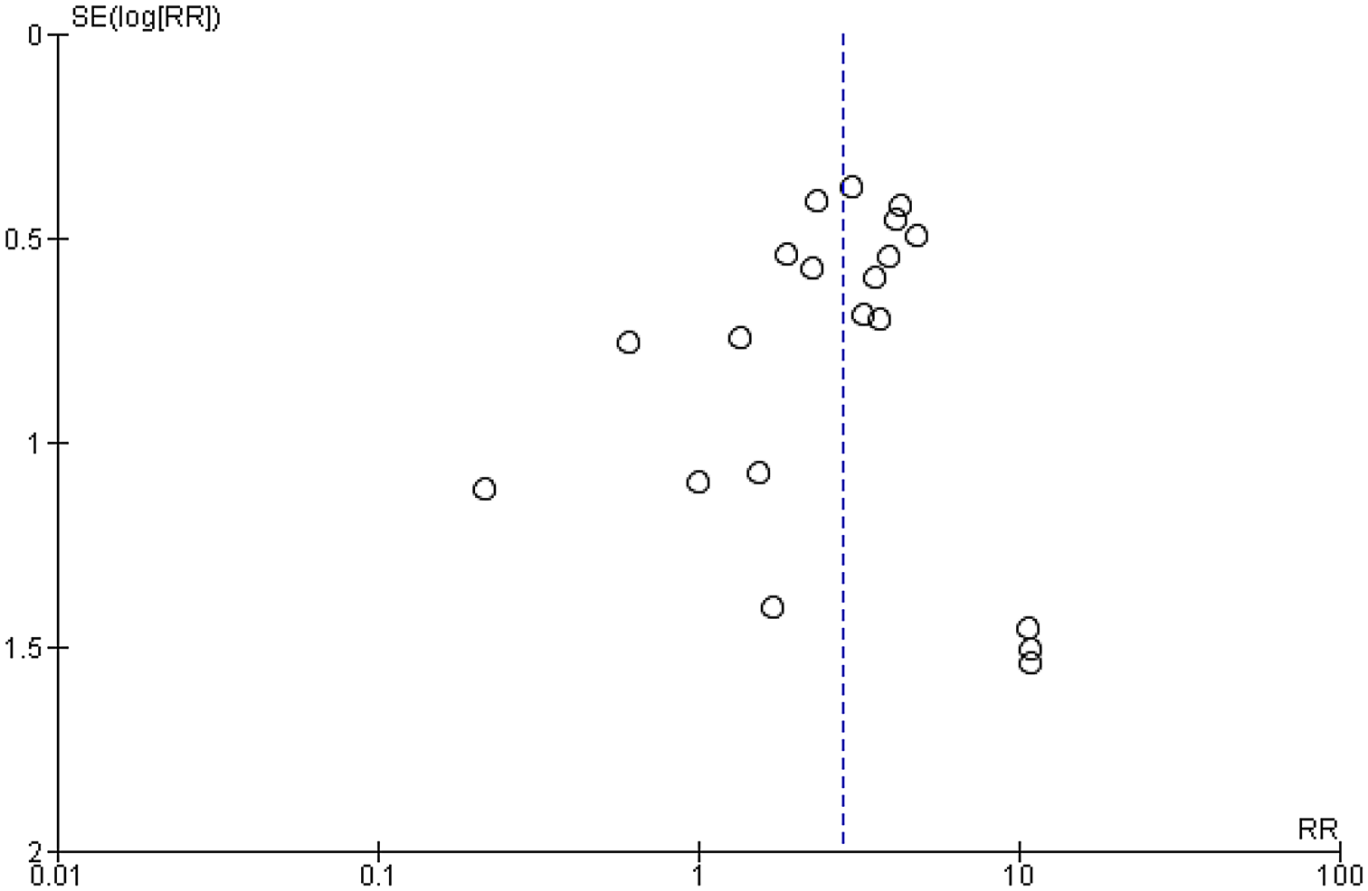
Funnel plot on publication of recurrence for primary versus patch repaired congenital diaphragmatic hernia patients.

Only a funnel plot of studies reporting recurrence could be made (Fig. 3), because more than ten studies could be included (see methods). Visual interpretation showed that there was no real asymmetry, indicating that small studies report on both positive and negative or no statistical significant effect of patches for this outcome.

### Sensitivity analysis

Sensitivity analyses were performed on the outcomes excluding those studies with ROBINS-I scored as serious or critical. For chylothorax, only three studies were of low or moderate risk of bias, however the risk remained significant (2.81 [1.09–7.26]. After analysis, the risk on recurrence remained significantly higher for patch repaired patients (risk ratio 2.86 [2.09, 3.93]), which was also the case for SBO (1.93 [1.02, 3.12).

## Discussion

This systematic review reports on the difference in rate of surgical complications, including chylothorax, recurrence and SBO for primary versus patch repaired CDH patients. This study shows that patch repaired patients have a significantly higher risk on developing several surgical complications. This review is the first to evaluate the evidence on surgical morbidity in CDH patients, as shown in the overview of the current literature in Table 1. However, confounding for severity of defect or disease was not possible, mainly due to lack of data in reporting on the severity of the CDH in the publications on surgical complications used in this study. After 2006, the CDH registry produced a standardized classification (A-D) to describe the size of the defect^45^. Unfortunately, still little studies report on this defect size specifically, therefore confounding for severity of size of defect and its morbidity is not possible neither performed for most outcomes^46^. Patch repaired patients are often the patients with the largest defects, which is why patch repair is often seen and used as a surrogate marker for severity of disease. Although there have been many studies and technological advances in treatment with use of patches, the mortality rate still remains higher than primary repaired patients (26–35% in patch repaired, versus 2.3–12.7% in primary repaired patients)^47,48^. However the severity of the disease is assumed to be linked with the size of the defect and subsequently the need for a patch to close the defect.

There are many types of patch material available. Although there remains ongoing discussion on the best type of material, most pediatric surgical centers use non-absorbable patches such as PTFE. The choice of material is also partly based on surgeon’s preference and the availability in the different centers^49^. Some state that there is no real significant difference in for example recurrence rate or SBO between different types of material^4,50^. A review by Puglandia et al.^6^ recommended to use PTFE oversized/dome shaped patches to reduce recurrence rate after inclusion of 213 abstracts, however, with a level of evidence of IV, due to the lack of any high level of evidence studies reporting on the most preferable type of material. They also stated that it could also be caused by the technical factors of patch repair^51^. The most mentioned disadvantages of PTFE are the inability to grow with autologous diaphragmatic tissue^52^ and formation of adhesions^39^. Also, as Tsai et al.^4^ stated complications may, to a greater extent, be due to technological factors rather than to the use of a patch itself. In this review, sub-analyses on type of patch material (and technological factors) were unfortunately not possible, due to the wide range of, not always specified, use in material in the studies.

### Haemorrhage

No studies could be included describing the difference in rate of haemorrhage for primary or patch repaired patients. However, intra-operative bleeding rate seems to be higher after closure with a patch compared to primary repair^40^. Haemorrhage in CDH patients could be related to use of anticoagulants (for example in ECMO patients). These ECMO patients are the most severely affected patients, presumably with larger defects, often requiring a patch. Therefore, the contribution of solely the patch itself is often hard to distinguish. The fact that haemorrhage is not often reported in CDH studies as a primary or secondary outcome could be due to the fact that amount of blood loss is mostly an estimation from the treating surgeon and due to the retrospective nature of most CDH studies, this variable was probably not available for most authors.

### Chylothorax

The incidence of CDH in surgically repaired patients with a chylothorax is 71%^53^. Our review clearly shows that patch repaired patients are at greater risk on developing a chylothorax. Chylothorax is considered a serious complication in these already high risk neonates leading to electrolyte abnormalities, malnutrition and increased risk of infections due to immunodeficiency. It can subsequently lead to increased respiratory morbidity with longer periods of oxygen supply and hospitalization^19,25,54^. However, confounding for the development of chylothorax could be ECMO treatment, which has been suggested to be a predictor as well^19^. Sensitivity- or sub analyses could not be performed due to the small number of included studies, lack of data and more importantly the variety of criteria for chylothorax used in the studies.

### Recurrence

Patch repaired patients had a higher risk on developing a recurrence, which is probably partly due to severity of disease. When correcting for approach of repair, the risk was not significant anymore for the MIS repaired patients, which could be caused by a selection bias in this group. Then again, different types of patch materials were used, for which confounding was not possible. There was a wide range of follow up in the studies, which was not always mentioned and, as a long term follow-up is relatively new in these patients could have influenced the incidence of recurrences^28^. However, of the 20 studies included for recurrences, 15 studies reported on length of follow-up, which was at least one year (Table 1), in which most recurrences are expected to occur^4,28,30^. Above that, the manner and timing of diagnosis of a recurrence (for example the inclusion of asymptomatic patients or standard chest radiograph in standard follow-up) was often not specified^37^.

### Small bowel obstruction

Ten studies reported on small bowel obstruction (SBO), divided by type of repair. Due to the wide range of follow-up (reported range 27 months to median 8.6 years as shown in Table 1^28,30,31,38–40^), the rate of SBO could be underestimated and might be higher after a longer follow-up^55^. Moreover, three studies did not report on the length of follow up^12,29,42^. Moreover, for this outcome specifically, it would be interesting to confound for type of patch material used, which could lead to different rates of SBO^30,39^. Whatever the cause, patch repaired patients have a higher risk on developing a SBO, which should be closely monitored and early detected.

The outcomes of this review demonstrate the fact that a clinically significant amount of CDH patients after surgery remains at risk on developing several (possibly severe) morbidities, especially after patch repair. This is why a close long-term follow-up in a multidisciplinary clinic is required, though sufficient data on the ideal length and frequency of visits of follow-up has not been found yet. This is due to the fact that most studies are single center studies and include small numbers of patients^56^. In this review the focus was mainly on short term complications in these patients. Whatever the exact cause of the difference in surgical morbidity between these two groups of patients, this review supports the advice to at least monitor (high risk) patients during the first period of life.

Limitations of this review are the low quality of the studies (for example design, mostly retrospective) which led to small sample sizes, also due to the incidence of the disease and numbers of missing variables. One of most important limitations was the inability to adjust for contributing or confounding factors, such as severity of disease, subtype of CDH and type of patch material, which led to a low quality of evidence according to the GRADE system (Table 2). Therefore, further research could influence the impact of the estimated effects^57^. Another bias could be different treatment strategies between both centers and surgeons, because use of patch is often an intra-operative risk assessment. Because there are small number of studies regarding this subject, studies published from 1990 (including patients treated from 1980 onwards) were included. However, as treatment strategies possibly confounding the outcomes might have been different, sub-analyses were performed, if possible, to adjust for the treating period. This showed that in recurrence and SBO, outcomes were comparable. Sensitivity analyses showed that the outcomes remained similar after exclusion of low quality studies. The only type of study to conclude whether patch repair itself is an independent risk factor for surgical complications, would be a randomized controlled trial (RCT) which is not feasible. An RCT would not be feasible neither ethical because the larger defects (type D) cannot be closed by primary repair. For the very small defects (type A) it would be ethically difficult to repair it with a patch if it were possible to primary repair the defect without the patch. The best possible scenario would be to randomize cases where both treatment options are plausible, e.g. intermediate defects (type B). This leaves a small subset of patients and may make a RCT non-feasible. However, although an RCT is not achievable, it does not undermine the fact that especially for patch repaired patients, treatment needs to be improved to lower the almost two times higher risk on surgical complications.

**Table 2:** Methodological quality according to the Grading of Recommendations Assessment, Development, and Evaluation (GRADE) system.

Quality of evidence (grade)^15^						Effect		Quality	Importance
Number of studies	Risk of bias*	Inconsistency	Indirectness	Imprecision	Other considerations	Relative (95% CI)	Absolute		
**Haemorrhage**									
0	–	–	–	–	–	–	–	–	–
**Chylothorax**									
5	Serious^1^	No serious inconsistency	No serious indirectness	Serious^2^	None	2.47 [1.25–4.87]	57 more per 1000 (from 10 to 149 more)	Low	Critical
**Recurrences**									
20	Serious	No serious inconsistency	No serious indirectness	Serious^3^	None	2.83 [2.16–3.71]	107 more per 1000 (from 68 to 158 more)	Low	Critical
**Small bowel obstruction**									
10	Serious	No serious inconsistency	No serious indirectness	Serious^3^	None	1.90 [1.31–2.76]	59 more per 1000 (from 20 to 116 more)	Low	Critical

*Scored according to *ROBINS-I tool*^16^.

^1^Some studies scored as serious or critical risk of bias (due to selection bias and inadequate follow-up, according to ROBINS-I tool).

^2^Due to wide range of confidence interval and ‘optimal information size (OIS)’ not met (downgrade one level).

^3^OIS not met (downgrade one level).

## Conclusion

Patch repaired congenital diaphragmatic hernia patients are at greater risk on developing surgical complications such as chylothorax, recurrence and small bowel obstruction. Current evidence is restricted to retrospective cohort studies and adjustment for morbidity severity is still not possible. Large prospective studies are required to collect adequate information to find the real causative factors. This could lead to new innovations that could minimize risk on surgical complications, especially in high risk CDH patients.

## Supplementary Information

Supplementary Information.

## Data Availability

All data generated or analysed during this study are included in this published article.
